# Breast milk jaundice affects breastfeeding: From the perspective of intestinal flora and SCFAs-GPR41/43

**DOI:** 10.3389/fnut.2023.1121213

**Published:** 2023-02-21

**Authors:** Huan Huang, Juan Huang, Wendi Huang, Nanqu Huang, Miao Duan

**Affiliations:** ^1^Department of Pediatrics, Third Affiliated Hospital of Zunyi Medical University (The First People’s Hospital of Zunyi), Zunyi, Guizhou, China; ^2^Department of Pharmacology and Chemical Biology, Shanghai Jiao Tong University School of Medicine, Shanghai, China; ^3^Key Laboratory of Basic Pharmacology of Ministry of Education and Joint International Research Lab of Ethnomedicine of Ministry of Education, Zunyi Medical University, Zunyi, Guizhou, China; ^4^National Drug Clinical Trial Institution, Third Affiliated Hospital of Zunyi Medical University (The First People’s Hospital of Zunyi), Zunyi, Guizhou, China

**Keywords:** breast milk jaundice, intestinal flora, short-chain fatty acids, G protein-coupled receptors 41 and 43, neonate

## Abstract

Breast milk jaundice (BMJ) is one of the main factors leading to interruption or early termination of breastfeeding. Interrupting breastfeeding to treat BMJ may increase the adverse consequences for infant growth and disease prevention. The Intestinal flora and metabolites are increasingly recognized as a potential therapeutic target in BMJ. First, dysbacteriosis can lead to a decrease in the metabolite short-chain fatty acids. At the same time, SCFA can act on specific G protein-coupled receptors 41 and 43 (GPR41/43), and a decrease in SCFA downregulates the GPR41/43 pathway, leading to a diminished inhibition of intestinal inflammation. In addition, intestinal inflammation leads to a decrease in intestinal motility and a large amount of bilirubin enters the enterohepatic circulation. Ultimately, these changes will result in the development of BMJ. In this review, we will describe the underlying pathogenetic mechanism of the intestinal flora effects on BMJ.

## Introduction

**Figure 1:**
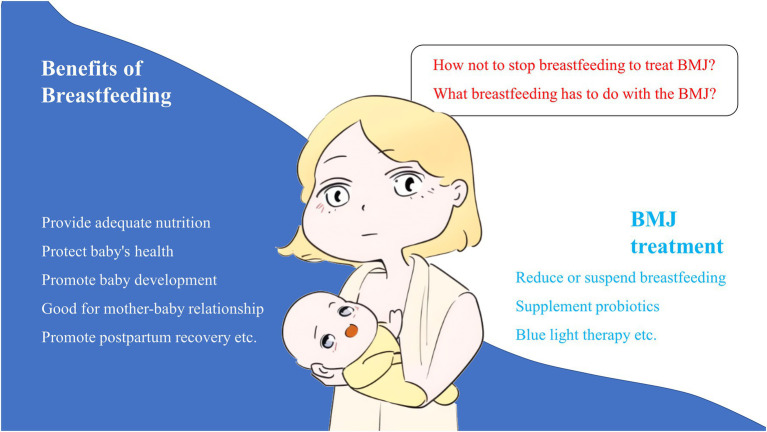
The benefits of breastfeeding and the main modalities for treating BMJ, as well as issues to consider.

Breastfeeding is considered as an optimal way to feed infants during the neonatal period, providing them with the developmental nutrients needed and shaping their immune systems (1). Most studies have shown that exclusive breastfeeding provides potential long-term benefits to neurodevelopment (2, 3). Interrupting breastfeeding to treat breast milk jaundice (BMJ) has long been controversial and may increase the risk of early termination of breastfeeding (4). In the community, the impact of neonatal BMJ on reduced breastfeeding and vaccination rates has been widely observed, which may resulting in adverse consequences for infant growth and disease prevention, even though clinicians generally advise continuing exclusive breastfeeding during BMJ (5). Therefore, clarifying the pathogenesis of BMJ is of great importance to prevent the occurrence of these adverse effects (Figure 1).

Breastfeeding jaundice and breast milk jaundice are two patterns of jaundice in newborns, which can occur with breastfeeding (6). The former frequently show excessive initial weight loss and be dehydrated in newborns who due to not receive sufficient breast milk causing early unconjugated hyperbilirubinemia between 4 and 7 days postpartum (7, 8). The latter usually can be appeared to infants who are well suckling, with satisfactory weight gain, and mild jaundice which typically occurs 1 week after birth, and possibly last up to 12 weeks, in addition, hyperbilirubinemia can be observed decreases when breast milk is replaced with infant formula (9).

The etiological mechanism of BMJ is not completely clear, and the increase in enterohepatic circulation is the usually compelling theory, although it may be caused by several factors. Notably, recent studies have reported that dysbacteriosis is one of the causative factors for BMJ and played an important role in the pathogenesis of BMJ (10, 11). In addition, studies have shown that the metabolites of microbiota also played an important role in physiological functions and have been regarded as a bridge between microbiota and diseases, such as bile acids, branched-chain amino acids, short-chain fatty acids, tryptophan, and indole derivatives (11, 12). Among these metabolites, short-chain fatty acids (SCFA) has become a focal point in recent years. Studies have shown that SCFA played an important role in maintaining the integrity of the intestinal epithelium and repairing the mucosa after injury (13, 14). However, few studies have focused on the role of SCFA in the pathogenesis of BMJ. The purpose of this review was to explore the value of intestinal flora and SCFA in the pathogenesis of BMJ. Reviewing and analyzing these mechanisms will help prevent and control BMJ, thereby ensuring the rate of breastfeeding.

## BMJ influences breastfeeding

The World Health Organization (WHO) recommends exclusive breastfeeding of infants for the first 6 months of life, but among the factors leading to interruption or early termination of breastfeeding, BMJ is one of the main factors (5). Notably, in recent years, with the popularization of breastfeeding in China, more and more infants are found to develop BMJ in the clinic, and BMJ is gradually becoming the leading cause of neonatal jaundice (15). Neonatal jaundice is a common condition that affects up to 80% of newborn babies and has become an important public health problem (16, 17). Accumulating evidence noted that breast-fed infants are at higher risk of jaundice than formula-fed infants (18, 19). Newman et al. (20) first reported that breastmilk was a contributing factor to the development of neonatal jaundice. Breastmilk contains the substance beta-glucuronidase, which, in the intestine, decouples glucuronic acid from conjugated bilirubin and allows bilirubin to be reabsorbed by the enterohepatic circulation (21). In addition, bilirubin undergoes selective metabolism by UDP-glucuronosyltransferase (UGT) 1A1, that *UGT1A1* gene mutation is closely related to BMJ (22, 23). BMJ is usually harmless and a self-limiting disease with a good prognosis for infants. However, few newborns with high levels of serum unconjugated bilirubin may damage the child’s central nervous system, leaving severe neurological sequelae (24, 25). Therefore, exploring the possible pathogenesis of BMJ can help relieve some of the anxiety of breastfeeding in pregnant women and help the health of newborns.

## Intestinal flora and BMJ

The human intestinal flora is composed of microbial communities that is known as a “microbial organ” within the host, a new human physiological system, and a “superorganism” with human tissues and organs (26, 27). These microbial communities are interconnected, engaged in a continuous exchange of information with the host cell, and regulated the transformation of important chemicals (28).

Studies have shown that dysbacteriosis is closely related to the occurrence of BMJ: Under normal conditions, certain bacteria in the intestinal tract can transform bilirubin into stercobilin and affect bilirubin excretion by promoting Intestinal motility during bilirubin metabolism (8). However, dysbacteriosis can lead to a decrease in bilirubin secretion, which result in higher enterohepatic circulation and thus induce BMJ (10, 29, 30). Moreover, *Lactobacillus rhamnosus GG* were used to treat jaundice without stopping breastfeeding, and more satisfactory results were achieved (31). As mentioned above, dysbacteriosis is closely related to the occurrence of BMJ, but the exact mechanism of how dysbacteriosis lead to BMJ remains to be further investigated.

## SCFA is the key between dysbacteriosis and the occurrence of BMJ

The intestinal flora effect on the host is mainly through metabolites, among them, SCFA has received extensive attention in intestinal diseases in recent years (32, 33). Interestingly, SCFA may be the key between dysbacteriosis and the occurrence of BMJ. SCFA are mainly produced by the fermentation of dietary fiber by microbiota, which is produced in the upper part of the colon near the ileocecal junction (34, 35). Coincidentally, the enterohepatic circulation of bilirubin that induced BMJ also happens to be hydrolytically separated at the end of the ileum, and then forms unconjugated bilirubin that is absorbed by the intestine (36). Therefore, the former and the latter are very close to the site of production and are relevant. In addition, Yang et al. found that bacteria reduction in bilirubin encephalopathy is associated with SCFA metabolism (37). Chen et al. showed that the abundance of some bacteria that mainly produced SCFA in the bilirubin encephalopathy group were significantly lower than that in the control group. Our study has also found that a significant difference in SCFA between the BMJ group and the control group (38). The decrease in SCFA due to dysbacteriosis may be an important reason for the occurrence of BMJ.

## SCFA may affect bilirubin secretion via the GPR41/43 pathway

**Figure 2:**
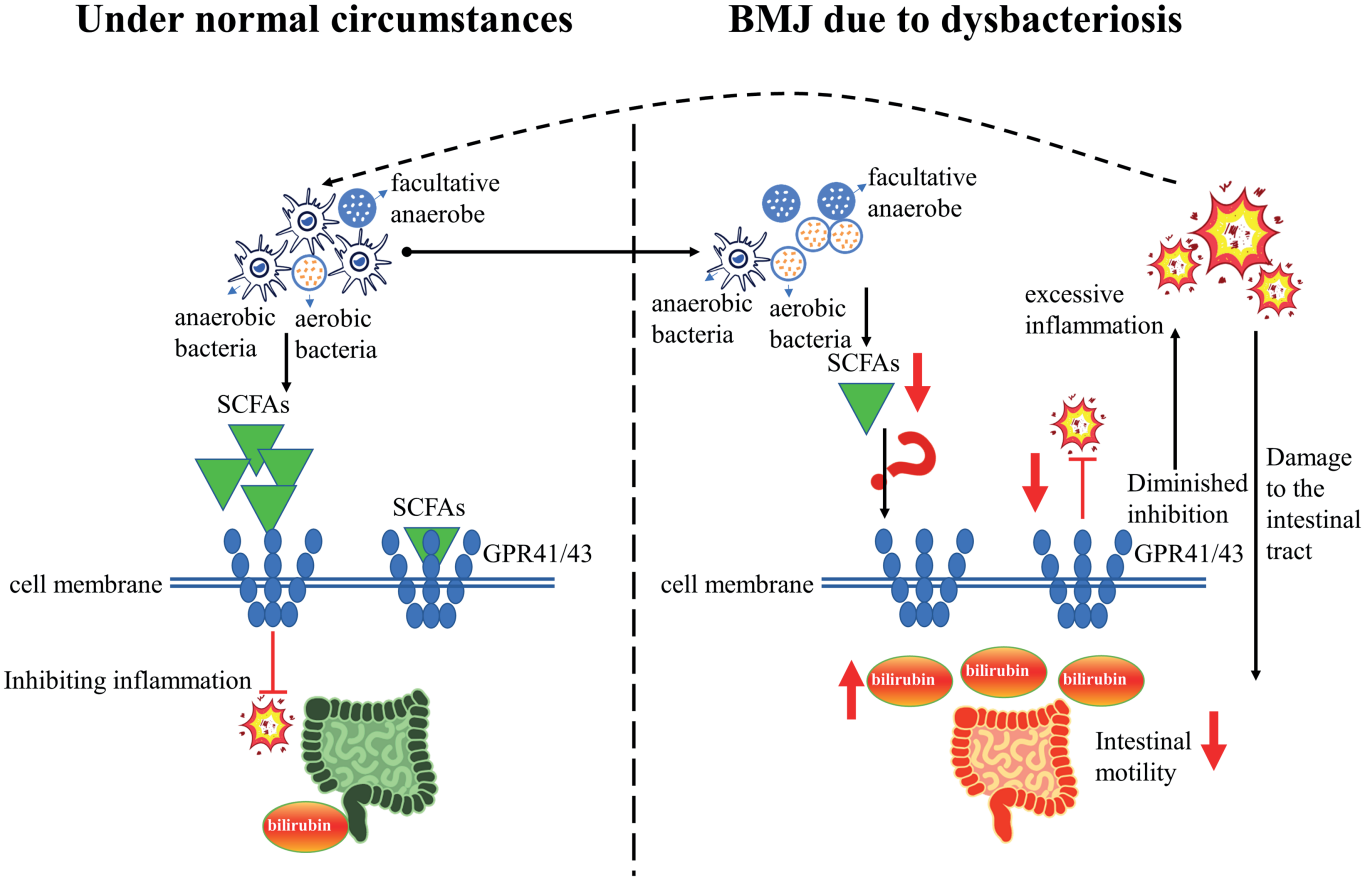
A simplified schematic diagram: dysbacteriosis leads to a decrease in SCFA, and downregulate the GPR41/43 pathway, resulting in the occurrence of BMJ.

The healthy intestinal tract is basically maintained in anaerobic or low-oxygen conditions, resulting in the composition of the intestinal flora being dominated by anaerobic bacteria (39, 40). Studies showed that compared to infants with formula feeding, a decrease in anaerobic bacteria represented by *Veronococcus* spp. and *Clostridium* spp. in infants with breastfeeding (41). Another study reported that an increased abundance of facultative anaerobe (predominantly *Proteobacteria*) in the intestinal flora of infants with BMJ (10). In infants with BMJ, there was an increase in aerobic *Streptococcus* spp. and a decrease in anaerobic *Enterococcus spp.* (42). This suggests that the composition of intestinal flora in infants with BMJ is mainly characterized by a change in anaerobic bacteria transform into facultative anaerobes and aerobes. This may be due to the alteration of intestinal flora induced by intestinal inflammation (43), which results in a decrease of metabolites represented by SCFA. SCFA can act on specific G protein-coupled receptors 41 and 43 (GPR41/43), thereby modulating the immune response (the main response presented is inhibition of inflammation) (44). A decrease in SCFA due to dysbacteriosis can aggravate the intestinal inflammatory response, which leads to a decrease in intestinal motility, resulting in a large of bilirubin entering the enterohepatic circulation and ultimately causing the development of BMJ (45, 46). Thus, dysbacteriosis affect bilirubin excretion may by regulating the SCFA-GPR41/43 pathway (Figure 2).

## Conclusion

An increasing large of studies have demonstrated that gut microbiota regulates bilirubin metabolism in enterohepatic circulation. Probiotics can affect neonatal hyperbilirubinemia *via* various potential mechanisms. A study demonstrated that *Bifidobacterium* can inhibit bilirubin enterohepatic circulation by reducing the activity of β-glucuronidase (47). *Saccharomyces boulardii* has been found a potential mechanism for decreasing enterohepatic circulation to, namely increase intestinal polyamines to promote intestinal maturityis (48). Prebiotics are non-digestible food supplement which decreased serum bilirubin *via* regulate intestinal microflora to remove the regulation of β-glucuronidase in the middle colon to reduce the enterohepatic circulation of bilirubin (49, 50). The relationship between intestinal microbiota and encephalopathy is an attractive topic, the Microbiota-gut-brain axis is a relatively new concept. Studies have demonstrated that the dysbiosis of gut microbiota can indirectly affects the blood brain barrier and brain regions *via* short chain fatty acid (51).

Dysbacteriosis can lead to a decrease in SCFA, and downregulate the GPR41/43 pathway, leading to a diminished inhibition of intestinal inflammation, thereby a decrease in intestinal motility and a large amount of bilirubin entering the enterohepatic circulation. Ultimately, these changes will lead to the occurrence and development of BMJ (Figure 2). Thus, modulating dysbacteriosis in BMJ is an effective strategic target for reducing serum bilirubin, but detailed information on the mechanisms remains unknown and this is an area where more research is needed. Although probiotics and prebiotics have a significant impact on the treatment of hyperbilirubinemia, the efficacy remains controversial. It is necessary to investigate the mechanisms in detail in order to provide compelling new therapeutic strategies for BMJ. In addition, more multicenter randomized clinical trial studies are needed to further elucidate the long-term benefits or risks of probiotics and prebiotics on BMJ. Overall, understanding the mechanisms of intestinal flora in the role of BMJ may increase the promotion and support of breastfeeding, and achieve the United Nations Sustainable Development Goal of maternal and child health.

## Author contributions

All authors listed have made a substantial, direct, and intellectual contribution to the work and approved it for publication.

## Funding

This work was supported by the National Natural Science Foundation of China (82260317), funds of the Zunyi Science and Technology Bureau (2018–10, 2018–190, and 2020–12), and Science and Technology Department of Guizhou Province (ZK [2021] 371).

## Conflict of interest

The authors declare that the research was conducted in the absence of any commercial or financial relationships that could be construed as a potential conflict of interest.

## Publisher’s note

All claims expressed in this article are solely those of the authors and do not necessarily represent those of their affiliated organizations, or those of the publisher, the editors and the reviewers. Any product that may be evaluated in this article, or claim that may be made by its manufacturer, is not guaranteed or endorsed by the publisher.
